# The Journey of Human Transthyretin: Synthesis, Structure Stability, and Catabolism

**DOI:** 10.3390/biomedicines10081906

**Published:** 2022-08-06

**Authors:** Chiara Sanguinetti, Marianna Minniti, Vanessa Susini, Laura Caponi, Giorgia Panichella, Vincenzo Castiglione, Alberto Aimo, Michele Emdin, Giuseppe Vergaro, Maria Franzini

**Affiliations:** 1 Department of Translational Research and New Technologies in Medicine and Surgery, University of Pisa, 56126 Pisa, Italy; 2 “Health Science” Interdisciplinary Research Center, Scuola Superiore Sant’Anna, 56127 Pisa, Italy; 3 Cardiology Division, Fondazione Toscana Gabriele Monasterio, 56124 Pisa, Italy

**Keywords:** transthyretin, TTR amyloidosis, retinol-binding protein, thyroxine, retinol, TTR clearance, ER-associated degradation pathway

## Abstract

Transthyretin (TTR) is a homotetrameric protein mainly synthesised by the liver and the choroid plexus whose function is to carry the thyroid hormone thyroxine and the retinol-binding protein bound to retinol in plasma and cerebrospinal fluid. When the stability of the tetrameric structure is lost, it breaks down, paving the way for the aggregation of TTR monomers into insoluble fibrils leading to transthyretin (ATTR) amyloidosis, a progressive disorder mainly affecting the heart and nervous system. Several TTR gene mutations have been characterised as destabilisers of TTR structure and are associated with hereditary forms of ATTR amyloidosis. The reason why also the wild-type TTR is intrinsically amyloidogenic in some subjects is largely unknown. The aim of the review is to give an overview of the TTR biological life cycle which is largely unknown. For this purpose, the current knowledge on TTR physiological metabolism, from its synthesis to its catabolism, is described. Furthermore, a large section of the review is dedicated to examining in depth the role of mutations and physiological ligands on the stability of TTR tetramers.

## 1. Introduction

Transthyretin (TTR) is a homotetrameric protein found in the plasma or serum and cerebrospinal fluid (CSF), mainly synthesised by the liver and the choroid plexus (CP). TTR was previously known as prealbumin because it migrated in front of albumin in serum protein electrophoresis [1]. Following the discovery of its role as a transporter of thyroxine (T4), the name was converted into “thyroxine-binding prealbumin”. It was only in 1981 that The International Union of Biochemistry converted the name into “transthyretin” (*trans*ports *thy*roxine and *retin*ol) [2], a name suggested when it was discovered that the protein was also able to bind both thyroxine and retinol complexed with retinol-binding protein 4 (RBP) [3]. Indeed, only a small fraction of circulating TTR (less than 20%) is involved in the transport of T4, which is mainly carried by thyroxine-binding globulin (TBG) and albumin [4], while most TTR tetramers are involved in the transport of holo-RBP. The association between TTR and RBP assumes considerable importance as it is necessary to avoid glomerular filtration and renal catabolism of RBP [5] due to the small size of holo-RBP (21kDa). Based on holo-RBP affinity for TTR and on their plasma concentrations (2 µM and 3.6–4.5 µM, respectively), it has been estimated that about 95% of circulating RBP is associated with TTR [6,7,8].

Plasma TTR levels rise after birth, reaching a concentration of 20–40 mg/dL in adults, and then TTR levels decrease after 50 years of age [9]. In clinical practice, TTR is considered a surrogate marker of diet adequacy since it is rich in the essential amino acid tryptophan and has a relatively short half-life (2.5 days) [10]. Indeed, a plasma TTR concentration below 10 mg/dL has been associated with malnutrition [11]. Nevertheless, plasma TTR is also negatively influenced by the acute-phase response due to inflammation, which is often associated with malnutrition. Thus, TTR levels can be helpful in evaluating and diagnosing a malnutrition status, but it is important to consider the involvement of inflammatory processes to interpret TTR levels correctly [12].

In the last years, TTR has gained considerable importance in the setting of amyloidosis, a protein misfolding disorder caused by the extracellular deposition of a β-sheet-rich protein as insoluble fibrils. Amyloid TTR (ATTR) amyloidosis is among the most common forms of amyloidosis in human pathology, and it can present as a hereditary form caused by TTR mutations (variant ATTR, ATTRv) or acquired ones due to the deposition of TTR wild-type (ATTRwt) [13]. The ATTRwt was previously known as “senile systemic amyloidosis” since the amyloid fibrils are mainly observed in elderly patients (>75 years) [14]. A few clinical studies have measured plasma TTR in patients with ATTRv or ATTRwt amyloidosis. These studies reported lower plasma TTR in patients with ATTRv amyloidosis as compared to reference values (18-45 mg/dL) [15,16] as well as low values, but still in the normal range, in ATTRwt amyloidosis [17,18].

TTR fibril formation has been investigated in many in vitro studies, which all agree that ATTR amyloid fibrils form because of tetramers dissociation into monomers which spontaneously misfold, forming amorphous aggregates, oligomers, and then amyloid fibrils [19,20,21,22]. The in vitro oligomerisation occurs in both ATTRv and ATTRwt amyloidosis; this process is faster in ATTRv amyloidosis, where the stability of TTR is compromised by genetic mutations [23,24]. The reason why TTRwt, despite the absence of destabilising mutations, also is intrinsically amyloidogenic in some subjects remains unknown. 

Given the clinical importance of TTR, in this review, we will discuss the current knowledge of TTR structure and metabolism, highlighting the variables that stabilise or destabilise the native protein in all stages of its life cycle, from synthesis to catabolism.

## 2. Tissue-Specific Regulation of Transthyretin Expression

Human TTR is a 127-amino acid (AAs) protein encoded by a single-copy gene mapped to chromosome 18q11.2-q12.1 [25]. The gene has a size of about 6.9kb and consists of four exons, three introns, a TATA box-like sequence at nucleotides 24–30, and a CAAT box-like sequence at nucleotides 95–101 [26,27]. The first exon contains 95 base pairs (bp) and 26 bp of 5’ untranslated region; it codes for a 20 amino acid signal peptide as well as the first 3 AAs residues of the mature protein. Exon 2 (131 bp) codes for AAs residues 4 – 47, exon 3 (136 bp) for residues 48–92, and exon 4 (253 bp) for residues 93-127 [26,28]. TTR mRNA encodes for the pro-TTR monomers (147 AAs), whose N-terminal region corresponds to a hydrophobic signal peptide of 20 AAs, which is cleaved to produce the native TTR monomer [29].

The cleavage of the signal peptide is necessary to free the first 9 AAs of the mature TTR monomers, which represent a structural “disordered region” [30] essential for monomer-monomer assembly into dimers, the first step in tetramer formation [31].

The liver and the CP are the most abundant and well-described sites of TTR synthesis in humans, but TTR expression has also been identified in a minor amount in the placenta, pancreas, yolk sac, and retinal pigment epithelium.

### 2.1. Liver and Choroid Plexus

The protein synthesised and released by the liver is the main source of TTR in plasma [32], where it circulates associated with T4 and holo-RBP. Thus, the physiological functions of hepatic TTR are related to the distribution of T4 and retinol throughout the body. 

The TTR gene expression in the liver is regulated at the transcriptional level and is controlled by hepatocyte nuclear factors (HNF) [33]. During the acute phase response, there is a significant reduction in the binding of HNFs to the *TTR* promoter, which correlates with a decrease in TTR expression [34]. Indeed, as a typical negative acute-phase plasma protein, the *TTR* gene in the liver is downregulated after trauma, inflammation, or malnutrition resulting in a marked decrease in circulating protein levels.

In the CP, the *TTR* gene is not subjected to acute phase negative regulation [35], suggesting it must be regulated independently of the liver *TTR* gene [36]. The structure of the *TTR* gene is identical in the two tissues: they have the same starting site for mRNA synthesis and the same enhancer sequences [37]; this suggests that the cell-specific distribution of transcription factors is responsible for tissue-specific expression of the gene [38]. In the liver, HNFs are the transcription factors involved in the activation of *TTR* gene transcription, but these factors are absent in the CP, where the specific identity of the transcription factors involved is yet to be understood. It is thought that in CP, the *TTR* gene is regulated by transcription factors that are closely related to the HNFs [38], which have the binding sequences in 3kbp of the 5’ flanking region [37].

Differences in gene regulation could be due to the important role of T4 in the brain. TTR synthesised by epithelial cells [39] of the CP is secreted into the cerebrospinal fluid (CSF) [40], where it is involved in the delivery of T4 to stem cells and progenitor cells within the brain, which requires T4 for the regulation of the cell cycle [41]. Since the adult and developing brain is sensitive to thyroid hormone effects, it is extremely important to maintain adequate levels of thyroid hormones even during trauma or inflammatory conditions when the reduction of plasma TTR and albumin results in a reduction of total circulating thyroid hormones [36]. Furthermore, TTR is the main carrier for T4 in the CSF [42], while in plasma, it carries only 15% of the whole T4 [43,44]; this further highlights the important role of TTR for the nervous system.

### 2.2. Placenta and Visceral Yolk Sac

The production of TTR by placenta trophoblasts plays a crucial role in foetal development in the first trimester [45]. Since the foetus is not able to produce its own thyroid hormone until 16 weeks of gestation, it must rely on the maternal T4 supply carried by placental TTR for brain development [46,47]. The presence of TTR has also been identified in the visceral yolk sac, supporting the idea of its importance in the active transport of T4 as well as retinol from the maternal circulation to the developing foetus [48]. The *TTR* gene regulation in these sites is poorly understood, but the transcriptional binding proteins CCAAT/enhancer binding protein (C/EBP) and activator protein-1 (AP-1), implicated in the transcription promotion of important genes during foetal development, could be involved [49,50]. 

### 2.3. Pancreas

TTR expression in the pancreas occurs mainly in pancreatic α-cells, whereas β-cells may produce TTR only at a low degree [51]. Gene regulation and function of TTR synthesised by these particular cells are largely unknown, but it seems that TTR promotes glucagon/insulin release and cell survival in both α [52] and β [53] cells. Low levels of plasma TTR have been found in patients affected by diabetes type I [4.4 µmol/L (24 mg/dL) compared to 5.3 µmol/L(29 mg/dL) in normal subjects], reinforcing the hypothesis that TTR may be associated with glucose homeostasis [54].

### 2.4. Retinal Pigment Epithelium

TTR is synthesised together with RBP by the retinal pigment epithelium of the mammalian eye [55,56]. These proteins may be involved in the delivery of all-trans-retinol to Müller and amacrine cells [57], where it is converted to retinoic acid required for photoreceptor functioning. Little is known about the distribution of TTR in the human eye. A recent study showed that TTR distribution in the vitreous directly correlates with that of retinal tissues, suggesting a local distribution or transport of TTR from the retina to the vitreous [58]. In studies on vitreous amyloidosis, ATTR appeared to result from locally synthesised protein from the retina [59,60].

## 3. TTR Structure

The X-ray crystal structure of human TTR was determined in the laboratory of Colin Blake in 1971 [61]. These structural studies provided information on both the active tetrameric conformation of TTR and the binding sites of both its ligands.

**Figure 1:**
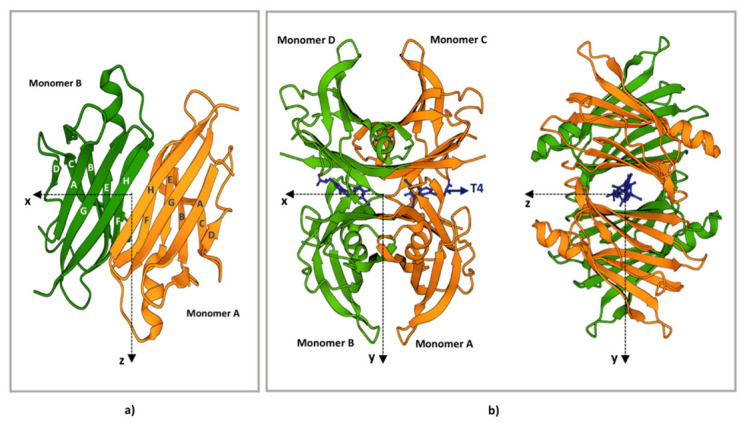
TTR structure. (**a**) Ribbon diagram of the AB dimer interface (**b**) Ribbon diagram of the TTR tetramers. The X-axis passes through the T4 binding channels, which are formed at the interface of monomers D-B and C-A. The halogen binding pockets (HBP) are shaped by the following amino acids: HBP1, Lys15-Leu17-Thr106-Val 121; HBP2, Lys15-Leu17-Ala108-Ala109-Leu110; HBP3, Ser117-Leu110-Thr119-Ala108. T4 is represented in blue. The figure has been produced using “www.rcsb.org”web site (protein ID code 1QAB), access date 22 June 2022.

Human TTR is a homotetrameric, slightly acidic (isoelectric point of 5.3) protein with a molecular mass of 55 kDa composed of four identical monomers, with a molecular weight of approximately 14 kDa [62]. Each monomer consists of eight β-strands structures designated with letters from “A” to “H” and one short α-helix of nine residues included between strands E and F. All strand interactions are antiparallel except for the interaction between strands A and G. These eight β-strands are connected by loops and are arranged in two groups of twisted β-sheets [44,63,64]. Interstrand hydrogen bonds allow the organisation of a tertiary structure of β-strands constituted by an inner (strands DAGH) and an outer (strands CBEF) β-sheet, which are orthogonal to one another [44,63,64] (Figure 1).

Two TTR monomers are arranged into dimers by hydrogen bonding between two F (F, F’) and two H (H, H’) strands from adjacent monomers. This provides an extensive contact region where the β-sheets are strictly packed in a layer-by-layer manner contributing to the stability of the molecules [44,63,64].

Two dimers then form a tetramer mainly through hydrophobic bonds between loops, including between the β-strands A-B and G-H, finalising the assembly of the globular TTR. Thus, the quaternary structure of TTR occurs in interactions between four identical monomers, but its conformation is maintained by the dimer–dimer interface. Indeed, the dimer is thought to be the basic structural unit of mature TTR. This perspective is strengthened by the observation that the contact region between dimers is smaller than those between monomers and consists of hydrophobic and hydrophilic interactions [44,63,64]. Thus, in physiological conditions, TTR is a globular homo-tetramer organised as a dimer of dimers. Even if the tetrameric structure is considered stable, a monomer exchange process has been shown in in vitro studies on recombinant proteins and consists of a slow disassembly of tetramers into TTR monomers followed by a quick reassembly [65].

## 4. TTR Structural Stability

In the assembling of the TTR tetramer, two cylindrical hydrophobic channels are generated at the dimer–dimer interface that can accommodate one T4 molecule each (Figure 1). The hydrophobic channel contains two pairs of three symmetrical sets of halogen binding pockets where the four iodine atoms of T4 are placed [61,66]. Even if the two channels are symmetric, there is a 100-fold difference in the binding constants for the first and the second T4 molecule. Indeed, because of negative co-operativity between the two sites, only one site can be occupied by T4 under physiological conditions [67]. Interestingly, each T4 binding site is formed by residues of two monomers belonging to opposite dimers (Figure 1), which become connected by T4 itself, leading to a drastic stabilisation of the native TTR tetramer. One of the current therapeutic strategies against ATTR amyloidosis is based on improving the kinetic stability of the native TTR tetramers by molecules able to bind the T4 pocket, thus preventing the early stage of TTR dissociation [68,69,70]. Guidelines for ATTR amyloidosis treatment [71] suggest the use of tafamidis [72] and diflunisal [73] as stabiliser drugs. A further drug, AG10, is under evaluation in clinical trials [74].

**Figure 2:**
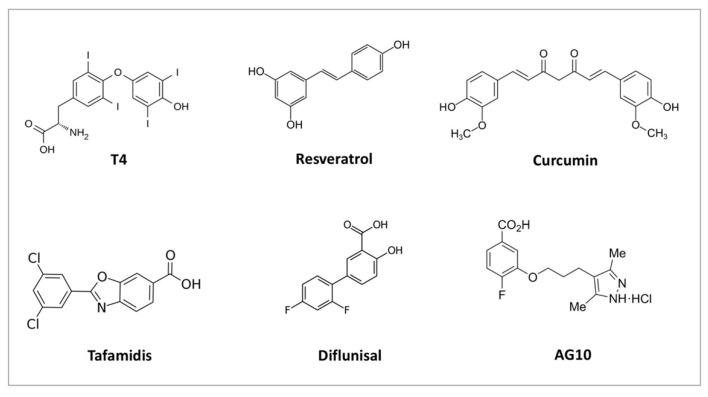
Chemical structure of some natural (T4, resveratrol, curcumin) and chemical ligands (tafamidis, diflunisal, AG10) able to bind and stabilise TTR tetramer.

In addition to T4, the central channel of TTR could bind a second endogenous ligand, the triiodothyronine (T3). However, the binding of T3 has a much lower affinity than T4, and circulating TTR binds virtually no T3 [43]. Several natural [75,76] and other chemical ligands [72,74,77] able to bind and stabilise the TTR tetramer have also been found (Figure 2); for reviews on these topics, see for example [78] and [79].

Contrary to T4, which is bound to the interior of TTR tetramers, holoRBP binds on its external surface, and each TTR tetramer has four putative binding sites for RBP, two in each dimer. 

**Figure 3:**
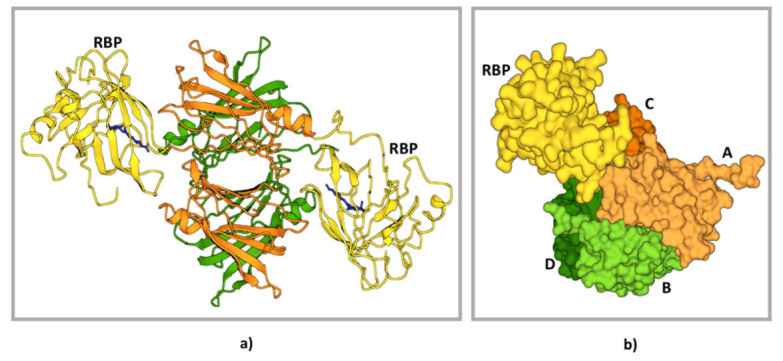
Structure of TTR–RBP complex. (**a**) Structure representation of the TTR–RBP complex. TTR: monomers A and C, orange; monomers B and D, green. RBP: yellow. Retinol: blue (**b**) Detail of the contact between the TTR subunits A, C, and D and the RBP molecule [left side, colour codes as in (**a**)]. Centre and right drawings show the interacting surfaces of RBP (centre) and of the TTR subunits A, C, and D (right). It is possible to appreciate how the RBP surface fits into a site formed by the arrangement of three TTR subunits (A, C, and D). The figure has been produced using “www.rcsb.org” web site, access date 22 June 2022.

The three-dimensional X-ray structure of the complex formed by TTR and RBP was first determined in 1995 by analysing crystals containing human TTR and chicken RBP [80]. Then, in 1999, the human TTR–RBP complex was resolved [81]. These studies showed that no more than two RBP molecules could be effectively bound because of their steric hindrance on the other two possible sites [80,82,83]. In humans, the two RBP molecules bind opposite dimers and are arranged in a 2-fold symmetry axis (Figure 3).

Both TTR and RBP contribute 21 amino acids to the protein–protein recognition interface, and most of these residues are in the C-terminal regions of the two proteins [81,84,85]. The RBP–TTR interface can be described with the three-dimensional docking model in which two complementary three-dimensional surfaces constitute the recognition interface. The two surfaces are outlined by amino acids from three TTR monomers and four distinct regions of RBP. The mainly involved amino acids in the interaction between one RBP, and TTR are within the following regions: for RBP 31-38, 63-67, 93-99, and 179-183; for TTR 82-90 and 114-115 for monomer A, 98-102 for B, 82-90, and 19-28 for D [81,85]. Interestingly, retinol is believed to be involved in RBP–TTR interaction since its hydroxyl end group is within hydrogen-bonding distance from the polypeptide chain of TTR [81,85]. Retinol binding induces conformational changes in RBP which strongly increases its affinity for TTR (holo-RBP, Kd 0.2 μM) compared to RBP alone (apo-RBP, Kd 1.2 μM) [7,86]. Accordingly, the stability of the TTR-holo-RBP complexes is reduced when retinol is removed; thus, the remaining TTR-apoRBP complexes are susceptible to degradation [3,87]. From this perspective, holo-RBP and not apo-RBP would exert a stabilisation effect on TTR tetramers. Interactions between TTR and RBP did not cause structural alterations in the proteins involved, but a subtle tertiary structural change was observed. Indeed a slight asymmetry in the extension of recognition surfaces between TTR and one or the other bound RBP was found [81,88]. The dissociation constants of the first and the second RBP molecules were investigated by mass spectrometry resulting in 1.5 × 10^–7^ M for the first bound RBP molecule and 3.5 × 10^–5^ M for the second one. It has been hypothesised that the binding of the first RBP induces a negative cooperative effect that decreases the affinity of TTR for the second RBP molecule [8].

The binding of T4 and holoRBP markedly increases the structural stability of TTR tetramers and consequently inhibits amyloid formation [89]. This possibility is supported by in vitro studies on TTR amyloidogenicity, which consider the independent and additive stabilising action of RBP and T4 on TTR tetramer disassembling [87]. The dissociation of TTR tetramers is decreased upon the formation of the TTR-holoRBP complex in a concentration-dependent manner. Indeed, monomers’ exchanges among TTR tetramers were significantly slowed by adding retinol together with RBP [87]. Furthermore, in the presence of holo-RBP and/or T4, the rate of TTR fibril formation was reduced by up to 50% even in the presence of L55P TTR, the most pathogenic TTR variant [87].

TTR stability can be negatively affected also by ageing, metal cations (especially Ca^2+^), and oxidative modifications [90] at the free cysteine residue in position 10 (Cys10), see later in the text. 

TTR proteolysis has recently attracted attention due to its implications for TTR instability and the pathogenesis of amyloidosis. It is challenging to identify the specific structural perturbation caused by proteolysis and how it affects the formation of subsequent dimeric or oligomeric intermediates [91]. Elucidating the detailed structural features of TTR dimers and oligomers is crucial since it may provide critical insights into the aggregation mechanism of TTR and help in identifying novel targets for therapeutic intervention. 

## 5. TTR Variants and Structural Stability

The dissociation of TTR tetramers results in partially unfolded monomers assembling into aggregates with various quaternary structures [90]. TTR aggregates undergo structural rearrangements forming cytotoxic oligomers. These dynamic, heterogeneous species of TTR represent intermediate states of early steps of ATTR amyloidogenesis [92,93]. 

The pathogenesis of ATTRwt is poorly understood, while it is largely accepted that the hereditary form is caused by autosomal dominant single point mutations in the coding region of the TTR gene that lead to the production of unstable TTR tetramers [94].

ATTRv amyloidosis was initially subdivided into “familial amyloid polyneuropathy (FAP)” and “familial amyloid cardiomyopathy (FAC)” based on amyloid tissue-selective deposition and pathology, but most of the variants were associated with a mixed phenotype, with varying degrees of neurological and cardiac involvement [95]. Recently, the International Society of Amyloidosis (ISA) has updated the nomenclature in favour of more exact definitions: FAC and FAP were renamed to ATTR amyloidosis associated with the specific causative mutation (e.g., ATTRV30M) and the accompanying main symptom (i.e., ATTR with cardiomyopathy) [64]. 

Among the known TTR variants (to date, more than 140) [96], only a small number have been described as non-amyloidogenic (e.g. G6S, H90N), and only three have been shown to form tetramers more stable than the wild-type one: T119M, R104H, and A108V are considered “trans-suppressor” mutations as they reduce the symptoms of the disease in heterozygous individuals carrying an aggressive mutation in the other allele [97,98,99]. Thr119 and Ala108 are both located in the T4 binding site: the methionine substitution for threonine 119 into methionine produces new hydrophobic contacts between the dimer-dimer interface, which increase the kinetic stability of TTR tetramers and the binding affinity for T4 [98]. The change of alanine 108 into valine induces a similar stabilisation action which determines a higher resistance to tetramer dissociation, although it does not induce an increase in the T4 binding affinity [97]. The arginine 104 is located in loops between strands F and G (F-G loop) at the surface of the TTR monomers [98]. The stabilising effect of R104H mutation seems to depend on thermodynamic stabilisation instead of kinetic stabilisation [98,99]: the final effect is an increase of TTR tetramer form at the expense of the misfolded monomers [99]. The greatest stabilising effect is associated with T119M and A108V variants, while R104H only modestly protects against aggregation [97].

**Figure 4:**
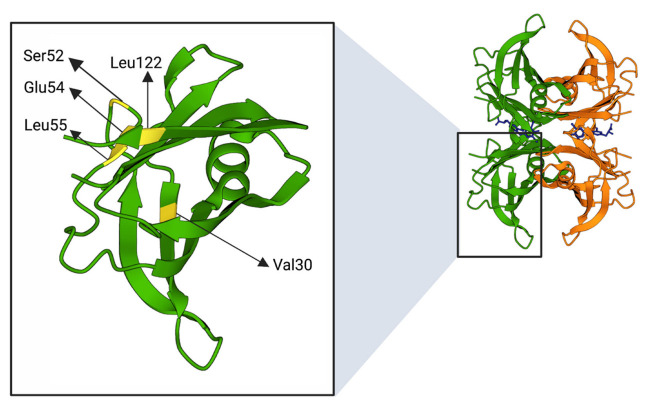
Positions of amino acids (highlighted in yellow) subjected to mutations described in Table 1. The figure was created through “www.rcsb.org” and “biorender.com” web sites (license agreement number KJ247URGZS), access date 22 June 2022.

Most of TTR mutations lead to a mature protein more susceptible to tetramer dissociation and monomers aggregation. The most studied TTRv are summarised in Figure 4. 

To obtain information regarding the structural changes responsible for the destabilisation of TTR tetramers, several researchers tried to solve the crystal structures of amyloidogenic TTR variants. The results derived from these X-ray crystallography studies showed almost identical structures between wild-type and mutated TTRs. Indeed, β-sheet tertiary structures of native TTR are minimally modified by pathogenic mutations [100,101]. Thus, X-ray studies could explain how single amino acid substitutions increase the TTR aggregation propensity only for those mutations highly affecting TTR structure [100,101]. 

A complementary approach to TTR structural studies has been provided by NMR, which allows the identification of minimal structural changes but is significant for kinetic and/or thermodynamic stability of tetramers and monomers, respectively [94]. One of the most important structural alterations that differentiate the structure of TTRv from TTRwt involves the EF-helix-loop region, positioned within the AB or CD dimer. This region seems to play an important role in TTR structural stability since any change in it affects the dimer–dimer interface and thus the stability of TTR tetramers [100,102,103]. Some information on structural alterations induced by amino acid substitutions is available only for the most known and well-studied pathological mutations, summarised in Table 1. For more details on TTRv, these web sources are recommended: gnomAD (https://gnomad.broadinstitute.org/) or Mutations in Hereditary Amyloidosis (www.amyloidosismutations.com; access date 10 June 2022)).

## 6. TTR Post-Translational Modifications

**Figure 5:**
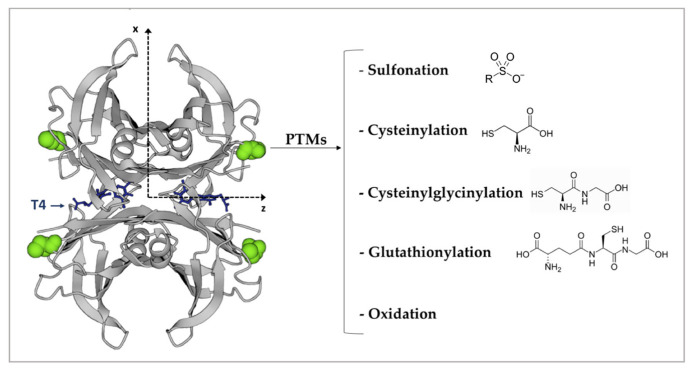
The four Cys 10 are highlighted in green and represented as a spacefill model. The most frequent Cys10 PTMs are indicated on the right. T4 is represented in blue. The figure has been produced by using “www.rcsb.org” web site (protein ID code 1QAB), access date 22 June 2022.

Post-translational modifications (PTMs) of proteins may be critical for the regular folding of the polypeptide chain, protein stability, and their normal turnover. Altered PTMs mechanisms may cause the structural destabilisation of proteins that form amyloid fibrils [111,112,113,114]. Even in the case of TTR, PTMs seem to participate in protein stabilisation. The most relevant and known PTMs for TTR occur at the free Cys10. Each TTR monomer contains a single Cys, which participates in the thyroid hormone-binding channels within the TTR tetramer; therefore, PTMs of Cys10 may interfere with the binding of thyroid hormones [115], thus indirectly affecting TTR stability. The strong reactivity of Cys10 is due to the fact that it is not involved in any intra- or inter-protein disulphide bond, which makes it susceptible to forming mixed disulphides with several thiol-reactive molecules, such as Cys, CysGly, and glutathione [116]. Other identified modifications on Cys10 are sulfonation and its conversion into organosulfur acids [117,118] (Figure 5). S-sulfonated or S-cysteinylated TTR are the most prevalent circulating forms, while only 10–15% remains unmodified at Cys-10 [117]. The type and grade of Cys10 modifications modulate TTR stability in different ways. S-sulfonation stabilises TTR tetramers [119,120], whereas S-cysteinylation enhances dissociation by 2-fold for the unmodified form [121]. Therefore, it is not surprising that Cys10 modifications are involved in tetramers destabilisation that triggers some forms of TTR familial amyloidosis [122,123]. These studies do not exclude that Cys10 modifications may also destabilise the unmutated protein in ATTRwt amyloidosis [121]. Furthermore, Met and Cys oxidation as well as carbonylation make TTR cytotoxic to human cardiomyocytes cell lines in a dose–responsive manner. Therefore age-related TTR oxidative modifications may play a role in the onset of ATTRwt amyloidosis [124].

## 7. TTR–RBP Complex Formation

Only a few studies were conducted to investigate the mechanism of RBP–TTR complex formation. It was initially proposed that the association between RBP and TTR occurred in plasma after the independent secretion of the two proteins [125]. However, the observation that TTR was accumulated within hepatocytes in vitamin A-deficient rats, resulting in decreased plasma TTR [126], led to the belief that RBP–TTR complexes may actually form inside the cells. Data supporting this hypothesis were obtained by Melhus and collaborators [127] using HeLa cells transfected both with RBP and TTR wild-type or modified with the endoplasmic reticulum (ER) retention signal peptide (KDEL). Authors showed that RBP could not be secreted when co-expressed with TTR–KDEL [127]. 

The importance of TTR in maintaining normal levels in mammals of plasma RBP, retinol, and thyroid hormones was confirmed in vivo in TTR knockout mice: animals were phenotypically normal and viable, but plasma levels of RBP, retinol, and thyroid hormones were significantly decreased compared to controls [128]. Furthermore, RBP–TTR complexes formation and secretion are likely sensitive to retinol supply [129]. Interestingly retinoids that could interact with RBP but, at the same time, prevented its association with TTR also inhibited RBP secretion [130]. These studies suggested that RBP–TTR complexes were formed inside the ER, as proved and confirmed in 1996 by Bellovino and collaborators [131]. 

## 8. Endoplasmic Reticulum Quality Control in TTRwt and TTRv Cellular Release

The critical role of ER in TTR synthesis has been confirmed by several studies conducted in the 2000s, concerning the mechanism by which TTR variants can bypass ER quality control systems [132,133,134,135,136,137,138]. This system aims to guarantee that only correctly folded or assembled proteins are translocated from the ER to their final destinations [139]. Misfolded or misassembled proteins are selectively retained in the ER by specific chaperone proteins or retro-translocated across the ER membrane and degraded by the cytosolic proteasome (ER-associated degradation, ERAD, mechanism) [140]. Therefore, ER quality control is a cellular protective mechanism to avoid the production of unstable proteins. However, some amyloidogenic proteins (including TTR) are stable enough to pass through the ER but misfold and aggregate once they reach their final destination [141]. 

Sekijima et al. tried to explain why almost all TTRv, despite their compromised folding energetics, can bypass the ERAD system showing a secretion efficiency similar to the TTRwt protein [138]. The authors analysed the secretion efficiency of 32 types of TTRv, finding that only the most destabilised variants, D18G [132] and A25T [133], exhibited a very low concentration in blood, suggesting a secretion defect by the liver [138]. Indeed, these TTRv are not associated with severe systemic amyloidosis. On the contrary, D18G and A25T seemed to be normally secreted by CP cells, and cause severe CNS amyloidosis [132,133]. The authors speculated that D18G and A25T are efficiently released by CP thanks to a high local T4 concentration which acts as a chaperone metabolite able to transiently stabilise TTR tetramers which can thus escape the ER quality control and be secreted into CSF. Once TTR is excreted into the CSF, the low local T4 concentration is insufficient to stabilise TTR that undergoes dissociation [132,133]. Therefore, ERAD protects against severe early-onset systemic amyloidosis, decreasing the secretion of most highly destabilised TTR variants. However, the mechanism is not able to prevent the secretion of those TTRv able to form tetramers stable enough to be secreted by the ER-assisted protein folding (ERAF) pathways, which include molecular chaperones and folding enzymes that allow proper folding and subsequent release from the ER of the newly synthetised proteins [142]. The ERAF is an error-prone process whose success depends on the combination of tissue-specific chaperones, metabolite chaperones (such as T4 for TTR tetramers) as well as thermodynamic and kinetic stability of the nascent protein. ER chaperone BiP and the protein disulfide isomerase PDIA4, key components of the ERAF mechanism, are differently involved in the secretory regulation of TTRv in various cellular models (HEK293T, HepG2, HeLa) [135,137]. 

The complex ER molecular networks, specific and unique for each tissue, with different distributions of protein chaperones, metabolite chaperones and osmolytes, could in part explain the tissue selectivity of TTR secretion and the organ tropism shown by TTR amyloidogenesis [138]. 

In addition to ER chaperones, some extracellular chaperones are detectable in body fluids, bind misfolded proteins and prevent their inappropriate protein-protein interactions and their aggregation into insoluble deposits [143,144]. Da Costa and collaborators have identified the extracellular chaperones probably involved in countering ATTR: haptoglobin, alpha-1-anti-trypsin, alpha-2-macroglobulin and clusterin, which plasma levels are highly increased in ATTR amyloidosis [136]. Increased levels of some of these extracellular chaperones have been identified for other amyloid diseases [145,146,147], supporting the hypothesis that their increase is necessary to compensate for the larger amount of altered protein prone to forming amyloid fibres [145].

## 9. TTR Catabolism 

### 9.1. Sites of Degradation

The first data about TTR catabolism were derived from studies conducted by Makover and collaborators in 1988 [148]. To determine the in vivo tissue sites of plasma and CSF TTR degradation, the authors utilised a nonmetabolisable tracer covalently linked to TTR that was administered to rats by intravenous or intraventricular injections. Upon tissue uptake, TTR underwent its normal degradation cycle, while the tracer was retained within cells in amounts proportional to the quantity of catabolised TTR. This tracer allowed the identification of TTR degradation sites and the estimation of the kinetics of TTR catabolism [148]. Tissue sites and quantitative patterns in TTR degradation were almost the same for the protein injected into the cerebrospinal fluid or plasma. No specific degradation of TTR was observed in the nervous system tissues, but, in both cases, the liver was the main organ involved in TTR degradation (36–38% of total body TTR degradation). Hepatocytes were the only liver cells involved in TTR degradation, while RBP was reported to be degraded in comparable amounts by both parenchymal and stellate cells [149]. In addition to the liver, TTR degradation mainly occurred in muscles (12–15%) and skin (8–10%), while kidneys, adipose tissue, testicles, and gastrointestinal tract catalysed about 1–8% of total TTR. Only less than 1% of TTR degradation occurred in other tissues. When the measured catabolic activities were normalised on tissue masses, kidneys, and liver resulted in being the most active organs [148]. 

On the bases of the mathematical model developed to interpret kinetic data, it was estimated that the entire CSF TTR pool was moved from CSF to plasma in 2.5–3.5 h, a time consistent with the turnover of whole CSF through the subarachnoid villi. The whole body TTR was then turned over in about 24 hours [148].

### 9.2. TTR Cellular Internalisation 

We know little about the TTR cellular uptake and its degradation mechanism. Sousa et al. [150] showed that renal uptake of TTR is mediated by megalin (also known as low-density lipoprotein-related protein 2, LRP2), which is a member of the LDL receptor family [151]. Megalin is a multi-ligand receptor expressed in the epithelium of kidney proximal tubes, where it is involved in the renal reuptake of plasma proteins, including free RBP [152]. Recent studies showed that TTR-internalisation is megalin-mediated in murine sensory neurons as well [153] and in the human placenta [49]. In the latter case, trophoblasts both secrete TTR into the maternal placental circulation and reuptake the protein preferentially as TTR-T4 complex after binding to maternal T4 [154].

In the liver, the most important site for TTR degradation, megalin is not expressed. Anyway, studies on human and rat hepatoma cell lines highlighted that also, in this case, TTR tetramers were endocytosed by a receptor-mediated process which resulted in being saturable (Kd between ~4 and ~10 nM vs. ~5 µM TTR plasma concentration) [154,155]. Interestingly, the complex TTR-holoRBP showed a 70% decrease in uptake in comparison to the TTR, while the uptake of the T4-saturated TTR was enhanced by 20% [154]. 

It was observed that the highly amyloidogenic L55P-TTR could not enter the cell line tested, while the mildly (V30M) and the non- amyloidogenic (T119M) TTR present a higher degree of internalisation than the wt proteins [154]. The differential rate of TTR clearance might play a role in the pathogenesis of ATTR amyloidosis.

Suosa and collaborators [156] showed that 1-2% of plasma TTR circulates bound to apolipoprotein AI (apoAI) of HDL lipoproteins. Therefore, the scavenger receptor class B type I (SR-BI) might be involved as TTR-receptor in the liver, but this possibility was then excluded [154]. Alternative candidates for TTR uptake were sought among other components of the LDL receptor family expressed by hepatocytes, i.e., the LDL receptor (LDLr) and the LDL receptor-related protein (LRP): experiments also excluded these two receptors [154]. However, TTR uptake was competitively inhibited by the receptor-associated protein (RAP), a common ligand for all the receptors of the LDLr family, including megalin [154]. These observations confirmed the existence of a receptor-mediated TTR internalisation in the liver and supported the hypothesis of some shared mechanisms between TTR and lipoprotein metabolism [154]. Nevertheless, the hepatocyte TTR receptor has not been identified yet.

### 9.3. Removal of TTR Aggregates 

TTR-related prefibrillar aggregates and amyloid fibrils are detectable in the extracellular matrix (ECM) of ATTR amyloidosis [157]. Several studies conducted on ATTRv and/or ATTRwt amyloidosis patients showed that TTR amyloid deposition manifests organ and tissue tropism, suggesting that the mechanism of TTR fibril deposition is closely related to the surrounding microenvironment [158]. Indeed, the specific composition of the ECM appears to play a key role in amyloidogenesis [159,160]. 

As previously mentioned, approximately 25% of the systemic TTR degradation occurs diffusely throughout the body (mainly from muscle and skin). Since fibroblasts are widely spread throughout the body and play a key role in maintaining the ECM, Misumi and collaborators [157] hypothesised that they could be the main cellular type involved in the TTR clearance in these sites. Indeed, fibroblast and macrophages can endocyte and degrade TTR aggregates in lysosomes both in vitro and in vivo, thanks to their migratory potential and their proximity to amyloid deposits in ECM [157]. Furthermore, fibroblasts may contribute to the degradation of TTR aggregates and fibrils by secreting matrix metalloproteinases (MMPs) into the ECM [157]. Further studies are needed to clarify the contribution of fibroblasts and macrophages in the clearance of TTR aggregates. Several authors are evaluating the possibility of stimulating phagocytosis by an antibody-related mechanism to promote amyloid reabsorption from tissues [161,162,163].

The follow-up of patients affected by severe forms of systemic ATTRv amyloidosis who underwent liver transplantation as curative therapy provided us with data on human turnover of tissue TTR amyloid. More in detail, liver transplantation in familial amyloidotic polyneuropathy patients was associated with a decrease in the total amount of amyloid deposits in abdominal fat tissues, but, interestingly, a change in amyloid composition was observed. Indeed, the ratio of wt-to-variant TTR shifted towards a greater contribution to the first [164]. This data suggested that remnant amyloid deposits of TTRv could represent a focal point for the deposition of the wild-type TTR (amyloid seeding) [165], furthermore showed that amyloid deposits underwent a turnover likely driven by dynamic processes of amyloid fibril formation and catabolism in the ECM. 

## 10. Conclusions

The journey of the TTR is depicted within this review, from its synthesis to tissue catabolism, with the aim to identify the key points of TTR metabolism likely involved in the onset and progression of organ amyloidosis. 

Many studies have been conducted both on cellular and animal models with the aim of clarifying TTR metabolism, but almost all pointed the attention to the TTR tetramer devoid of its physiological ligands, which, however, are essential for TTR stability. Thus, there are many knowledge gaps to be filled to translate experimental data into human pathophysiology. This research effort will provide a better understanding of the pathogenic mechanism of ATTR amyloidosis, especially of ATTRwt amyloidosis.

**Figure 6:**
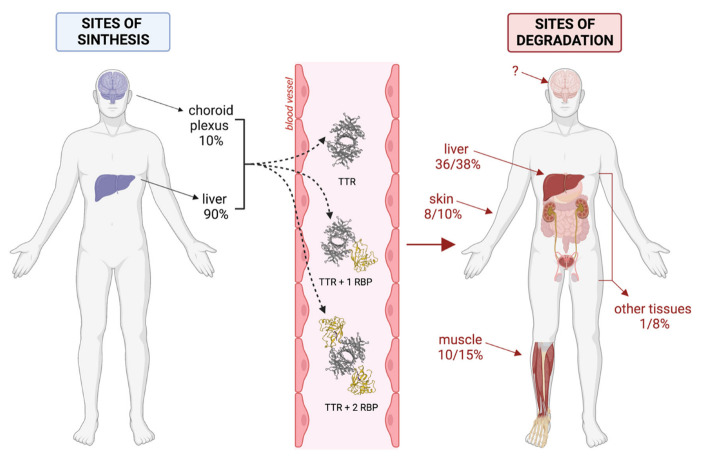
Reconstruction of the possible life cycle of TTR. The figure was created through “biorender.com” web site (license agreement number DL2489YBZI), access date 22 June 2022.

The main pieces of information available up today about TTR turnover have been summarised in Figure 6. Both in animal and cellular models, it was shown that RBP could be secreted only if complexed with TTR [126], although it is not clear whether in a 1:1 and/or 2:1 ratio. In hepatocyte cellular models, the secretion of TTR tetramers has been described [31,138], and their presence in circulation cannot be excluded. By the way, numerous experiments in animal models, including those for studying the clearance of TTR, were conducted by injecting the tetrameric TTR protein devoid of its ligands. These studies confirmed the possibility that TTR can subsist in plasma by itself. However, TTR tetramers might be cleared much more efficiently than the RBP–TTR complex by hepatocytes [154], so the balance between TTR and RBP–TTR complexes in plasma cannot be foreseen. The clearance of TTR has been studied only for the tetramers [148], so it is not known if the presence of RBP could actually change the rate of clearance. Anyway, it is of interest to observe that the main organs and tissues involved in TTR tetramers clearance (i.e., liver, muscle, skin) are not among those critically affected in ATTR amyloidosis (i.e., heart and nervous system).

A further point to be evaluated regards the role of retinol in the stability of RBP–TTR complexes; indeed, when absent, the affinity of RBP for TTR decreases; thus, it should be of interest to know if apoRBP–TTR complexes are still stable or if TTR tetramers are released [7,86]. 

Many in vitro studies investigated the process leading to the metamorphosis of TTR from the globular (physiological) to the fibrils (pathological) structure [165]. All these studies focused on how TTR tetramers are modified, but the role of RBP or T4 in preserving TTR function in vivo was scarcely investigated. The time has come to translate all this knowledge to a more physiological context that would allow us to determine what conditions favour the increase of free TTR tetramers which is the starting point of the amyloid cascade.

## Figures and Tables

**Table 1:** Main TTR gene mutations and related structural modifications.

TTRv (Other Name) *	Mutated Nucleotide (mRNA)	Clinical Phenotype	Involved Secondary Structure [30]	Overall Structural Alteration	Ref.
Val30Met (p.Val50Met)	c.148G>A	AN, E, LM, PN	β-strand B	Destabilisation of B and E strands resulting in a distortion of T4 binding channel. Causes a lower affinity for T4	[104,105]
Ser52Pro (p.Ser72Pro)	c.214T>C	AN, H, K, PN	β-bend	Stability alteration of C-D loop in protein monomer	[106]
Glu54Lys (p.Glu74Lys)	c.220G>A	AN, H, PN	β-strand D	Lys54 destabilises tetramer structure due to increased electrostatic repulsion between Lys15 of two monomers. The T4 binding pocket is markedly narrower in Glu54Lys compared with wtTTR, suggesting a decrease of affinity for T4	[107]
Leu55Pro (p.Leu75Pro)	c.224T>C	AN, E, H, PN	β-strand D	Disruption of hydrogen bond interaction between β-strands A and D leads a β-strand D structure highly disordered with different contacts between the subunits as well as a significant variation in the CE region of the monomer	[108,109]
Val122Ile (p.Val142Ile)	c.424G>A	H	122-127 AAs terminal loop	small changes in the region associated with the intra- and inter dimer interactions	[110]

* The name of TTRv is reported according both to the traditional and to the Human Gene Organization (HUGO) nomenclature which include the 20-AA signal peptide, e.g.: Val30Met (p.Val50Met). AN = autonomic neuropathy; E = eye; H = heart; K = kidney; LM = leptomeningeal; PN = polyneuropathy.

## Data Availability

Not applicable.
